# Do unbiased people act more rationally?—The case of comparative realism and vaccine intention

**DOI:** 10.1098/rsos.220775

**Published:** 2023-02-01

**Authors:** Kamil Izydorczak, Dariusz Dolinski, Oliver Genschow, Wojciech Kulesza, Pawel Muniak, Bruno Gabriel Salvador Casara, Caterina Suitner

**Affiliations:** ^1^ Faculty of Psychology in Wroclaw, SWPS University of Social Sciences and Humanities, Ostrowskiego 30b, 53-238 Wroclaw, Poland; ^2^ Social Cognition Center Cologne, University of Cologne, Koln, Nordrhein-Westfalen, Germany; ^3^ Warsaw Faculty, Centre for Research on Social Relations SWPS University of Social Sciences and Humanities, Wroclaw, Poland; ^4^ Department of Developmental and Socialization Psychology, University of Padova, Padova, Veneto, Italy

**Keywords:** realisms, self–others comparisons, unrealistic optimism, meta-analysis, vaccine intention, COVID-19

## Abstract

Within different populations and at various stages of the pandemic, it has been demonstrated that individuals believe they are less likely to become infected than their average peer. This is known as comparative optimism and it has been one of the reproducible effects in social psychology. However, in previous and even the most recent studies, researchers often neglected to consider unbiased individuals and inspect the differences between biased and unbiased individuals. In a mini meta-analysis of six studies (Study 1), we discovered that unbiased individuals have lower vaccine intention than biased ones. In two pre-registered, follow-up studies, we aimed at testing the reproducibility of this phenomenon and its explanations. In Study 2 we replicated the main effect and found no evidence for differences in psychological control between biased and unbiased groups. In Study 3 we also replicated the effect and found that realists hold more centric views on the trade-offs between threats from getting vaccinated and getting ill. We discuss the interpretation and implication of our results in the context of the academic and lay-persons' views on rationality. We also put forward empirical and theoretical arguments for considering unbiased individuals as a separate phenomenon in the domain of self–others comparisons.

## 1. Do unbiased people act more rationally?—The case of comparative realism and vaccine intention

Since the first outbreak of COVID-19, societies have faced ongoing uncertainty regarding health and life. Describing and understanding how we process this situation is a crucial task for social and behavioural science. Furthermore, the pandemic provided a unique opportunity to further our knowledge about basic socio-cognitive processes.

An exceptional challenge comes with (re)appraising the role of rationality in the face of such an unforeseen, mass-scale threat. At the earliest stages of the pandemic, it was not unusual to find opinions from prominent psychologists warning the public about excessive panic (see [1]). This 'irrational’ reaction was supposedly a distortion, a consequence of our cognitive biases, such as 'probability neglect’ [2] or our shortcomings in 'risk literacy’ [3]. Just a few months later, the reach and severity of the pandemic deemed the previously 'unreasonable overreaction’ a necessary measure (social distancing, mask-wearing, lockdowns, etc.).

This poses the question: what is a ‘rational’ or ‘irrational’ reaction to threat and to what extent is ‘debiasing’ societies desirable?

The present paper presents a mini meta-analysis of a series of multi-lab studies and two pre-registered follow-up studies, examining the prevalence, possible roots and consequences of comparative realism—a lack of optimistic or pessimistic bias in the estimations of COVID-19 contraction risk. Specifically, we investigate how this bias relates to COVID-19 vaccine intention. By tackling the issue of vaccination, we test whether the absence of comparative bias might be related to more rational behaviour in the face of COVID-19 threat.

### 1.1. Comparative risk assessments

According to social comparison theory [4], people have an innate drive to evaluate themselves. In most cases, they do it by evaluating their own achievements, abilities and traits in comparison with others (usually their peers). Similarly, when people estimate the probability of different events, they have the tendency to ‘believe that negative events are less likely to happen to them than to others, and they believe that positive events are more likely to happen to them than to others' [5, p. 807]. For example, people believe they are less likely to experience heart disease, divorce or a railway accident than their peers [5–8]. This common bias is called *comparative optimism* (CO) or *unrealistic optimism*. One of the prominent perspectives among social psychologists is that positive illusion helps people to cope with potentially threatening situations [9]. Some theorists postulate that positive illusions reduce stress and anxiety [10,11] or help people to retain a sense of personal control [12,13].

However, contrasting empirical evidence points to a negative association between comparative optimism and self-protective behaviours. For example, smokers who demonstrated comparative optimism were less likely to quit smoking, and more likely to perceive cigarettes as non-harmful [14]. Moreover, in a longitudinal study, college students who were comparatively optimistic about alcohol problems were more likely to experience them in the future [15].

Interestingly, some empirical studies found circumstances under which people hold a pessimistic bias. For example, Dolinski *et al.* [16] examined reactions among Polish citizens immediately after their exposure to nuclear radiation following the Chernobyl disaster. They found that the majority of participants believed that they were more likely to suffer radiation-related health problems than their peers—they displayed *comparative pessimism* (CP). A similar pattern of results was obtained by Burger & Palmer [17] in a study conducted after the 1989 California earthquake.

Comparative pessimism comes with possible benefits—in the aforementioned study by Dolinski *et al.* [16], those who exhibited pessimism were more likely to engage in self-protective behaviours.

### 1.2. Comparative realism and the goal of the present research

Summing up, optimism and pessimism are two possible outcomes of social comparisons. If individuals predict more favourable outcomes for themselves than for others, they are comparatively optimistic. If they assume more negative outcomes for themselves, they are comparatively pessimistic. As we have seen, depending on the situation, holding an optimistic or pessimistic bias can have positive or negative consequences (e.g. [15,16,18]). Strikingly, the majority of research has neglected to consider the third mode—*comparative realism* (CR).

Comparative realism can be defined as predicting one's own outcomes as similar to others' outcomes. This category has rarely been analysed in the literature (for an exception, see [19]), often confounded with comparative pessimism (e.g. [20]). We argue that this might be an important omission.

When comparative optimism is measured, there are usually multiple scale points that indicate various levels of pessimism and optimism and just one possible score that would indicate realism. Despite this, the few researchers who consider realism as a mode of thinking discovered a significant fraction of CRs (19% [20], 9.3%–56.2% [21]). Such a point-inflated distribution is common in many domains of health or environmental science [22–24] and can signal a twofold mechanism of the phenomenon: one mechanism accounts for the difference between the inflated score and other scores; the second mechanism accounts for the variance among the rest of the scores.

The number of cigarettes smoked weekly can serve as an example. If we examine this variable among the general population, we will obtain a large fraction of ‘zeroes’, as there are many non-smokers. Besides the inflated ‘zero’, we might expect a variety of scores, which will indicate the different patterns of smoking. Note that in such a case, the difference between 1 and 2 cigarettes per week is mathematically equivalent to the difference between 0 and 1. However, these differences are practically and theoretically non-equivalent. The first difference indicates a level of engagement and the second one marks the qualitative cut-off point between engagement and non-engagement.

The question arises as to whether there could be a qualitative difference between individuals who exhibit some degree of optimistic/pessimistic bias and individuals who do not exhibit it at all. In this article, we present evidence that such a qualitative difference not only exists but is relevant for health-related decision making.

The goal of the present research is to examine the role of comparative realism in vaccine intention and to identify psychological dispositions and cognitive processes related to comparative realism.^1^

## 2. Study 1: mini meta-analysis of the relationship between comparative bias and vaccine intention

To investigate the relationship between realism and constructive coping strategies in the context of the COVID-19 pandemic, we re-analysed six previously conducted studies that assessed: (i) comparative bias and (ii) COVID-19 vaccine intentions.

### 2.1. Method

When analysing a series of the authors' own studies that all share similar variables, it is advisable to combine the evidence in the form of a mini meta-analysis [25]. Such a strategy allows formal, statistical conclusions based on combined evidence and provides more precise estimates of effect size.

#### 2.1.1. Included studies

We included six studies from various populations (table 1), conducted between 4 June and 14 August 2020. These studies were part of a multi-laboratory research programme regarding comparative optimism and contained multiple variables measuring attitudes, beliefs and behaviours related to psychological functioning during the COVID-19 pandemic.

**Table 1.** Summary of the studies included in the mini meta-analysis*.*

nr	nationality	sampling source	time	*N*	comparative optimists	comparative realists	comparative pessimists
1	German	local online panel	10.07.20–22.07.20	129	61 (47.3%)	39 (30.2%)	29 (22.5%)
2	Italian	social media	05.07.20–16.07.20	100	68 (68%)	22 (22%)	10 (10%)
3	American	M-Turk	22.07.20	181	100 (55.2%)	34 (18.8%)	47 (26%)
4	Polish	students at a local university	05.08.20–14.08.20	565	253 (44.8%)	256 (45.3%)	56 (9.9%)
5	Polish	students at a local university	05.07.20–19.07.20	440	195 (44.3%)	189 (43%)	56 (12.7%)
6	American	Prolific	04.06.20	994	574 (57.7%)	263 (26.5%)	157 (15.8%)
				2409	1251 (51.9%)	803 (33.3%)	355 (14.7%)

Across six studies, we measured comparative bias by examining the estimation of getting COVID-19 for the self with the estimated risk for an average citizen (Study 6) and for both the average citizen and similar peers (Studies 1–5).

To examine the magnitude of comparative optimism and pessimism, we introduced a comparative index score (*C*_index_). This score is computed as the difference in risk estimations between ‘Self’ and ‘Others’—Positive *C*_index_ scores indicate comparative optimism (CO), whereas negative scores indicate comparative pessimism (CP). A *C*_index_ equal to zero indicates comparative realism (CR). In the case of all studies, the *C*_Index_ was recoded into a three-level categorical variable (CO, CR, CP).

In the first five studies, the comparative bias was measured by the same three questions, which were always translated into the native language of our target sample:

Risk_Me_: *How likely is it that you will become infected with coronavirus (SARS-CoV-2/COVID-19)?*
Risk_Peer_: *How likely is it that your average friend, or your average neighbour, will become infected with coronavirus (SARS-CoV-2/COVID-19)?*
Risk_Coutrymen_: *How likely is it that your average fellow-countryman will become infected with coronavirus (SARS-CoV-2/COVID-19)?*

All the aforementioned questions were answered on a 1 (absolutely impossible)–11 (quite certain) Likert-like scale.

From these questions, the *C*_Index_ was calculated, using the following formula:

$$C_{\mathrm{Index}}=({\mathrm{Risk}}_{\mathrm{Peer}}-{\mathrm{Risk}}_{\mathrm{Me}})+({\mathrm{Risk}}_{\mathrm{Coutrymen}}-{\mathrm{Risk}}_{\mathrm{Me}}).$$

In Study 6, comparative bias was measured on two levels, using Risk_Me_ and Risk_Coutrymen_, so the formula was: C_Index_ *=* (Risk_Coutrymen_ − Risk_Me_)

**Figure 1.**
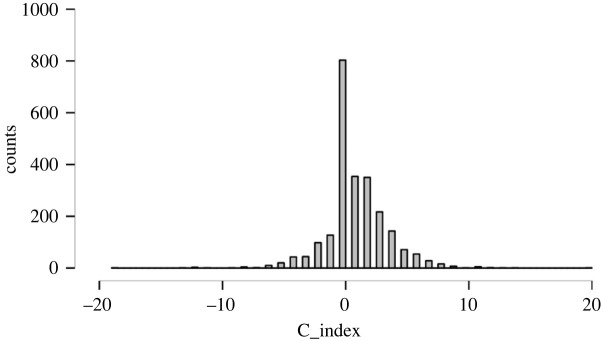
Distribution of *C*_Index_ across six studies (*n* = 2409).

In all six combined studies, we identified 51.93% of ‘comparative optimists', 33.33% of ‘comparative realists’ and 14.73% of ‘comparative pessimists' (figure 1).

#### 2.1.2. Variables

In all studies, the participants were asked the same question regarding their intention to vaccinate against COVID-19:

I will take the vaccine for the coronavirus/SARS-CoV-2 once it becomes available.

Participants provided their answers on an 11-point scale (1 = *absolutely impossible*, 11 = *quite certain*).

It is worth noting that at the time of data collection for Study 1, the SARS-CoV-2 vaccine was not yet available in any of the participants' countries, so the question about vaccine intention was hypothetical.

#### 2.1.3. Analysis

We conducted three separate mini meta-analyses using vaccine intention as a dependent variable and three comparisons between three comparative types as grouping variables: CR versus CO, CP versus CO and CR versus CP.

For each of the six studies, we extracted the effect size (rank-biserial correlation), standard error of effect size and sample size. To analyse our data, we performed a random-effect meta-analysis, using REML estimation.

All analyses were conducted in JASP v. 0.14.1 [26]. Databases are available along with the described analysis (https://osf.io/skc5d/).

### 2.2. Results

**Figure 2.**
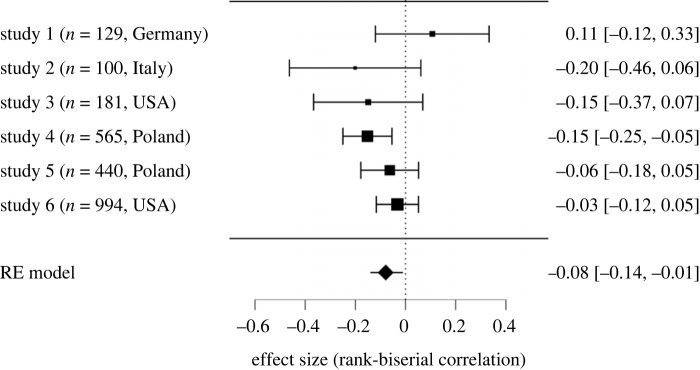
Forest plot—difference in vaccine intention between comparative realists (CRs) and comparative optimists (COs).

CRs were less eager than COs to vaccinate for COVID-19 (figure 2): *M*_CR_ = 6.36, s.d._CR_ = 3.40; *M*_CO_ = 6.98, s.d._CO_ = 3.06. The meta-analytic correlation was *r*_rb_ = −0.08 and the Wald test yielded significant results, *z* = −2.38, *p* = 0.017. Analysed effects proved to be homogeneous: *Q* = 7.18, d.f. = 5, *p* = 0.207, *τ*^2^ = 0.00, 95% CI [0.00, 0.06], *τ* = 0.04, 95% CI [0.00, 0.25], *I*^2^ = 26.69%.

**Figure 3.**
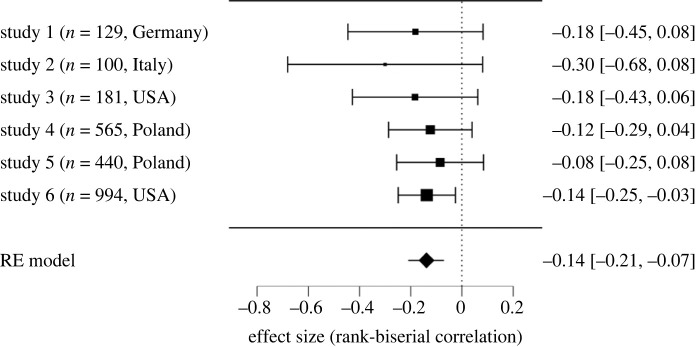
Forest plot—difference in vaccine intention between comparative realists (CRs) and comparative pessimists (CPs).

As shown in figure 3, a reluctance by realists was also found in the comparison with pessimists: *M*_CR_ = 6.36, s.d._CR_ = 3.40; *M*_CP_ = 7.38, s.d._CP_ = 2.78. Meta-analytic rank-biserial correlation was *r*_rb_ = −0.14, the Wald test yielded significant results, *z* = −3.74, *p* < 0.001. Analysed effects proved to be homogeneous: *Q* = 1.33, d.f. = 5, *p* = 0.931, *τ*^2^ = 0.00, 95% CI [0.00, 0.01], *τ* = 0.00, 95% CI [0.00, 0.10], *I*^2^ = 0.00%.

We did not find a significant difference between CPs and COs (meta-analytic *r*_rb_ = 0.08, Wald's *z* = 1.87, *p* = 0.062). Analysed effects proved to be homogeneous: *Q* = 7.69, d.f. = 5, *p* = 0.174, *τ*^2^ = 0.00, 95% CI [0.00, 0.09], *τ* = 0.06, 95% CI [0.00, 0.30], *I*^2^ = 31.86%. See electronic supplementary material for forest plot.

### 2.3. Discussion

Our meta-analysis indicated that realists displayed the lowest vaccine intention, with pessimists displaying the highest intention. The finding that pessimists are most likely to engage in active, preventive behaviours corresponds with previous research on nuclear risks [16] and current research on COVID-19 that reported a negative correlation between optimistic bias and vaccine intention [27]. But contrary to the aforementioned studies, the relationship is not linear. Moreover, when we try to interpret this relationship assuming that the optimistic bias is a simple, continuous variable with realism as a middle point, we encounter serious difficulties—pessimists did not significantly differ from optimists, and realists were less willing to vaccinate than both biased groups.

Although the effects were small, they are nevertheless theoretically and practically important. Theoretically, our effects were contrary to predictions and thus deserve attention. Practically, small effects can have impressive consequences when viewed at the population level [28]. Moreover, our small findings may prove important for understanding vaccine hesitancy, which is among the greatest threats to global health [29].

The fact that realists were the most vaccine-hesitant group is somewhat unexpected and to the best of our knowledge, there are no hints in the previous literature that would suggest such a phenomenon. One could argue that realists might have a lower vaccine intention because they perceive lower absolute risk levels than both biased groups. However, this explanation cannot account for our data, because there is no significant difference between realists, optimists and pessimists in terms of average risk estimations: the meta-analytical estimate coefficients were not significantly different from ‘0’ for both the comparisons between ‘realists’ and ‘optimists’ (meta-analytic *r*_rb_ = 0.01, Wald's *z* = 0.42, *p* = 0.676) and ‘realists’ and ‘pessimists’ (meta-analytic *r*_rb_ = 0.04, Wald's *z* = 0.92, *p* = 0.359) (see electronic supplementary material for plots and detailed analyses).

Another explanation for the difference in vaccine intention between realists and both biased groups pertains to their level of engagement in responses. Realism could be an artefact rooted in low-effort responses. That is, less motivated participants may have clicked all the risk levels (for ‘self’, ‘peer’ and ‘citizen’) in the same manner to finish the survey more quickly. However, we did not find a significant difference in the time spent on the survey. The completion time information was available in four out of six studies and it did not differ between realists and optimists (*U* = 124618.00, *r*_rb_ (993) = 0.01, *p* = 0.752) or between realists and pessimists (*U* = 29209.00, *r*_rb_ (604) = 0.01, *p* = 0.837)

To further the understanding of the detected differences, we assessed two follow-up, pre-registered studies with the aim to test possible explanations for lower vaccine intentions among realists. The first follow-up study examined the role of locus of control [30,31] and desirability for control [32].

## 3. Study 2: relationship between ‘realism’ and vaccine intention—the role of locus of control and desirability for control

A prominent view in the literature suggests that comparative optimism may be rooted in the sense of psychological control (e.g. [33]). The relationship between control and intention to vaccinate against COVID-19 can be rooted both in a cognitive or motivational perspective, namely the belief or the desire to be in control of one's health.

These two perspectives can match two psychological constructs, namely locus of control and desire for control. *Locus of control* (LoC) refers to how much control a person feels they have over their own actions. People with internal locus of control believe they have personal control over their behaviour [30]. *Desire for control* (DfC), on the other hand, is defined as the extent to which individuals are ‘motivated to feel as if they are in control of the events in their lives' [32, p. 148].

There is evidence indicating that both LoC and DfC are related to optimistic bias. On the one hand, a meta-analysis indicates that individuals who perceive more control over an event are more likely to be optimistically biased when asked about the chances of this event [12]. Moreover, Hoorens & Buunk [34] demonstrated that high-school students with a higher internal locus of control are more likely to display CO in relation to health problems.

Likewise, several studies found that different aspects of psychological control are related to vaccine intentions [35–37].

Given that sense of personal control is positively related to optimistic bias, we assumed that realists will have a lower internal locus of control and desirability for control than optimists. Furthermore, since psychological control proved to be related to vaccine intention, we predicted that LoC and DfC are good candidates for mediators of the relationship between optimistic bias and vaccine intention.

In Study 2, we assessed the degree to which high internal LoC and DfC accounts for the relation between comparative optimists and willingness to vaccinate.

### 3.1. Method

We pre-registered two hypotheses (https://osf.io/5csr9):

H1: Realists have a lower sense of personal control over pandemic situations than comparative optimists.
H2: Personal locus of control mediates the relationship between categorical *C*_Index_ (realists/optimists) and vaccine intention. Realists will have lower vaccine intention and a lower personal locus of control.

#### 3.1.1. Sample size justification, participants

We aimed to recruit a sample that would allow for meaningful statistical inferences concerning ‘realists’. A meaningful inference was defined as obtaining 80% power to detect an effect size of *d* = 0.2 with an alpha level of 0.05. We chose an effect size of *d* = 0.2 because in Study 1 the average effect size for the difference between realists and optimists in vaccine intention was *d* = 0.19. We decided to treat the effect size identified in the mini meta-analysis as the minimal effect size of interest because our empirical results were the only known rationale for our prediction. Besides these results, we had no other reason to expect any effect in this direction. Indeed, theoretical predictions would suggest an effect in the opposite direction. For that reason, we decided that (i) finding any effect in the same direction as in the mini meta-analysis would be theoretically interesting, and (ii) since we did not have enough resources to search for any minimal effect, we planned to search for an effect that was most plausible, judging by our latest empirical data.

An *a priori* power analysis for two independent groups and a one-tailed test indicated that we needed at least 310 participants per group. Based on previous research, we estimate that 33% of the population consists of realists, so we decided to recruit 1000 participants and then check whether we obtained the desired 310 realists.

Unfortunately, 1000 participants proved to be insufficient, as the percentage of realists turned out to be lower. Thus, we decided to recruit an additional 400 participants, obtaining 275 CRs (19.59%), 1013 COs (72.15%) and 116 CPs (8.26%). Although we did not reach the desired number of realists, resource constraints forced us to end the sampling.

The final sample consisted of 1404 participants across 65 nationalities (652 males, 747 females, 1 non-disclosed and 4 missing answers, *M*_Age_ = 24.63, min._Age_ = 18, max._Age_ = 65). Detailed information on sample demographics is available in the electronic supplementary material (https://osf.io/fndjc). Please note that, since we calibrated the power of our study to be enough to detect meaningful effects with respect to comparisons with realists, it would not be enough to detect analogical effects when it comes to pessimists (the least numerous category). For that reason, we did not conduct any analyses concerning pessimists.

As pre-registered, we excluded the participants who did not match our screening criteria, namely those who were either vaccinated against COVID-19 or had been officially diagnosed with this disease in the past. Besides these two filters, we allowed the panel to source participants from all available countries, without any quotas on demographic characteristics.

#### 3.1.2. Procedure

Data were collected via an online questionnaire through *Prolific* from 31 May to 15 June 2021.

After providing informed consent, participants were asked the pre-screening questions regarding the vaccination and COVID-19 infection, then about their vaccine intention. Next, they answered a block of questions diagnosing locus of control, desirability for control and comparative bias (in a randomized order). All questions used a ‘forced response’ option, which made proceeding to the next question impossible unless the participant provided a response for the current one. The demographic data were delivered by the Prolific panel.

The study was approved by the local ethics committee. The questionnaire in the .qsf and .pdf file is available in the electronic supplementary material (https://osf.io/mc23e).

#### 3.1.3. Variables

##### 3.1.3.1. Comparative bias

Comparative bias was assessed via three questions:

Risk_Me_: *How likely is it that you will become infected with coronavirus (SARS-CoV-2/COVID-19)?*
Risk_Peer_: *How likely is it that your average friend, or your average neighbour, will become infected with coronavirus (SARS-CoV-2/COVID-19)?*
Risk_Coutrymen_: *How likely is it that your average fellow-countryman will become infected with coronavirus (SARS-CoV-2/COVID-19)?*

All the aforementioned questions were answered on a 1 (absolutely impossible)–11 (quite certain) Likert-like scale. The answers were provided on a slider scale, with the default position as ‘1’.

The magnitude and the direction of comparative bias were calculated, using the following formula: *C*_Index_ *=* (Risk_Peer_ − Risk_Me_) + (Risk_Coutrymen_ − Risk_Me_). *C*_index_ was then recoded into three categories, Those with *C*_index_ = ‘0’ were categorized as comparative realists (CRs), those with positive *C*_index_ were comparative optimists (COs) and those with negative *C*_index_ comparative pessimists (CPs).

##### 3.1.3.2. Vaccine intention

The intention to get vaccinated was measured with the item: ‘I will take the vaccine for the coronavirus/SARS-CoV-2’.

Participants provided their answers on an 11-point scale (1 = *absolutely impossible*, 11 = *quite certain*). The answers were provided on a slider scale, with the default position as ‘1’.

Participants were also asked to briefly justify their answer in an open text box.

##### 3.1.3.3. Locus of control

Locus of control was measured with the brief version of Levenson's ‘locus of control scale’ [31]. The questionnaire consisted of nine statements which were evaluated by participants on a 7-point rating scale (1 = *strongly disagree*, 7 = *strongly agree*). The scale is divided into three subscales: internal control (e.g. ‘My life is determined by my own actions'; Cronbach's *α* = 0.63), ‘chance’ (e.g. ‘To a great extent, my life is controlled by accidental happenings'; Cronbach's *α* = 0.60) and ‘powerful others’ (e.g. ‘I feel like what happens in my life is mostly determined by powerful people’; Cronbach's *α* = 0.72). The score for all subscales was computed as a sum of ratings on all items.

##### 3.1.3.4. The desirability of control

We measured desirability of control with the ‘desirability of control scale’ [32] which consists of 20 7-point statements (1 = *the statement does not apply to me at all*, 7 = *the statement always applies to me).* A sample item is: ‘I prefer a job where I have a lot of control over what I do and when I do it’. (Cronbach's *α* = 0.79). The score was computed as a sum of ratings on all items.

### 3.2. Results

R programming language [38] was used to transform the data and JASP v. 0.14.1 [26] was used for statistical analysis. All analysis scripts are available at the OSF (https://osf.io/skc5d/).

The distribution of categories regarding the C_Index_ was: CRs, 19.59% of the sample; COs, 72.15%; and CPs, 8.26%. This distribution corresponds with the distribution obtained in the mini meta-analysis.

Also, the main effect discovered in the meta-analysis was confirmed as CRs had significantly lower vaccine intentions than COs: *U* = 126173.50, *r*_rb_ (1288) = −0.09, *p* = 0.008. Mean vaccine intention for CRs was *M* *=* 8.62, s.d. = 3.28. For COs it was *M* *=* 9.38, s.d. = 2.56. The visualization of distributions are available at the OSF (https://osf.io/fndjc).

#### 3.2.1. Confirmatory analyses

To test the first pre-registered hypothesis (i.e. comparative realists have a lower sense of personal control over the pandemic situation than comparative optimists), we conducted an independent samples comparison with the LoC ‘internal control’ subscale as a dependent variable and categorical *C*_Index_ (CRs/COs) as a grouping variable. Distribution of *C*_index_ proved to significantly deviate from the normal distribution; for that reason, we decided to use non-parametric statistics. Parametric analyses are available at the OSF and they yield the same conclusions.

A Mann-Whitney test yielded non-significant results = 142273.00, *r*_rb_ (1288) = 0.02, *p* = 0.709. An additional Bayesian analysis provided evidence in favour of the null hypothesis. Using zero-centred Cauchy's prior distribution with scale parameter *λ* = 0.2, we obtained a Bayes Factor in favour of the null hypothesis, *BF*_01_ = 2.67, which means that our data were two times more probable under the true null hypothesis. Conventionally, this result should be interpreted as ‘anecdotal’ evidence in favour of the null hypothesis [39]. The robustness analysis indicates that in order to obtain conclusive evidence (BF > 6), the prior scale should be *λ* > 0.52.

**Figure 4.**
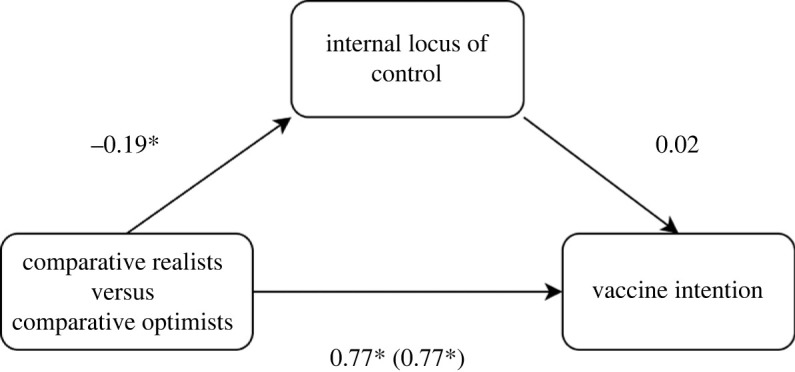
Path plot for mediation model with ‘Vaccine intention’ as the dependent variable, categorical CIndex (CR versus CO) as a predictor and Internal Control as the mediator.

Also, our second hypothesis (i.e. personal locus of control mediates the relationship between categorical *C*_Index_ (CRs/COs) and vaccine intention; realists will have lower vaccine intention and lower personal locus of control) was also disconfirmed. The bootstrapped mediation analysis (1000 replication, biased corrected percentile, *ML* estimator) indicated that while there is a significant total effect (*b* = 0.77, s.e. = 0.19, *p* < 0.001) and a direct effect (*b* = 0.77, s.e. = 0.19, *p* < 0.001) of categorical *C*_Index_ on vaccine intention, no significant indirect effect of personal locus of control is present (*b* < 0.00, s.e. = 0.01, *p* = 0.522). See figure 4 for a summary of the mediation model.

Additionally, we decided to test whether the results for our hypotheses would change if we used slightly different ways of distinguishing between comparative realists and comparative optimists. We tested three alternative variants. In the first, we used more liberal criteria to identify realists. Instead of *C*_index_ = ‘0’, we defined CRs as *C*_index_ between ‘−1’ and ‘1’ and COs as *C*_index_ > 1. In the second variant, we computed *C*_index_ using only ‘Risk_Me_’ and ‘Risk_Peer_’ − *C*_index_ = Risk_Peer_ – Risk_Me_’. In the third variant, we computed *C*_index_ using only ‘Risk_Me_’ and ‘Risk_Countrymen_’ − *C*_index_ = Risk_Countrymen_ – Risk_Me_’.

All three alternative analyses yielded the same conclusions—the hypotheses were not confirmed. The analyses can be found in the OSF folder (https://osf.io/skc5d/).

#### 3.2.2. Exploratory analyses

The previous analyses ruled out that internal locus of control explains the relationship between realism and vaccine intention. Thus, we explored whether any specific dimension of locus of control is related to vaccine intention or comparative bias.

We found that neither the *powerful others* nor the *desirability for control* subscale differs between CRs and COs. Only for the *Chance* subscale did the two groups differ significantly. That is, realists had higher ratings on the *chance* subscale, *U* = 128276.00, *r*_rb_ (1288) = −0.08, *p* = 0.024.

Neither the *chance* nor the *powerful others* subscale mediated the relationship between categorical *C*_Index_ and vaccine intentions. Moreover, from all examined control-related variables, only one was related to vaccine intentions. Vaccine intentions correlated negatively with the *powerful others* subscale of *locus of control: r*_1404_ = −0.06, *p* = 0.028.

#### 3.2.3. Qualitative analyses

In order to analyse participants' open answers, structural topic models were used with the stm package [40] of the software R [38]. The structural topic model assumes that documents are produced from a mixture of topics. Topics are then generated from a distribution of words. Based on these assumptions, stm generates topics of correlated words and assigns to each document a proportion of each topic. The function *textProcessor()* was used to clean the text. In order to decide the number of topics to extract, the fit of 30 models (from 1 to 30 topics) was compared. The best solution was chosen based on the highest held-out likelihood [41]. The output favoured a model with 21 topics. After that, using the function *estimateEffect()*, we tested how vaccine compliance and realism affected the prevalence of each topic. Interestingly, the prevalence of five topics was negatively affected by vaccine intentions:

(1) Side-effects (*B* = −0.005, s.d. = 0.001, *p* < 0.001). Example: ‘I'm still concerned about the possible side effects’.
(2) Distrust (*B* = −0.003, s.d. = 0.003, *p* = 0.003). Example: ‘I don't trust the hurried development of it, it does not guarantee any immunity and I won't let anyone put an experimental thing inside my body’.
(3) Side-effects due to time-related issues (*B* = −0.003, s.d. = 0.001, *p* = 0.002). Example: ‘I am not convinced of this vaccine as its testing was short. I want to see if people who are currently vaccinated will suffer (or not) from the vaccine’.
(4) Time-related worries (*B* = −0.002, s.d. = 0.001, *p* = 0.007). Example: ‘I don't trust a vaccine that was developed in such a short period of time’.
(5) Side-effects 2 (*B* = −0.008, s.d. = 0.001, *p* < 0.001). Example: ‘Unsure about the side effects so I am hoping to wait to see how it is going to be’.

Additional analyses indicated that the first three topics were more prevalent among realists compared with ‘biased’ participants (*B1* = 0.01, s.d.*1* = 0.005, *p* = 0.05; *B2* = 0.02, s.d.*2* = 0.006, *p* = 0.006; *B3* = 0.016, s.d.*3* = 0.006, *p* = 0.005).

Moreover, five other topics were positively associated with vaccine intentions:

(1) Trust in science (*B* = 0.003, s.d. = 0.001, *p* < 0.001). Example: ‘I believe in science’.
(2) Solution to the pandemic situation (*B* = 0.005, s.d. = 0.001, *p* < 0.001). Example: ‘In my opinion it is the only way to control the situation and protect the population’.
(3) General support for vaccination through trust in the country (*B* = 0.003, s.d. = 0.001, *p* < 0.001). Example: ‘Because in my country we have good medicine support’.
(4) Vaccine as a solution for affiliation needs (*B* = 0.003, s.d. = 0.001, *p* < 0.001). Example: ‘I want to take the vaccine so I can hug my friends and family again without the fear of making them sick’.
(5) Vaccination to protect others (*B* = 0.002, s.d. = 0.001, *p* < 0.001). Example: ‘I need to be as protected for this as possible in order to take care of my loved ones'.

In particular, the first topic was more prevalent for realistic participants (*B* = 0.022, s.d. = 0.007, *p* = 0.002).

### 3.3. Discussion

Despite the clear prediction substantiated by theory and previous research, personal locus of control proved to be unrelated to comparative optimism. While this result comes as a surprise, there are hints in the existing literature as to why it might have occurred. In the aforementioned meta-analysis of relationships between comparative optimism and sense of control [12], the authors identified an important moderator of the effect—exposure to risk. Among those who were less risk-exposed, the relationship between control and comparative optimism was significant, but among those who were at high risk of exposure, the relationship was not present. It might be the case that in the COVID-19 pandemic, we all feel highly threatened, which hampers the relationship between comparative optimism and sense of control.

Another explanation for this result is that while general, dispositional locus of control or desirability for control might be unrelated to comparative bias regarding COVID-19 infection, a sense of control over COVID-19 infection, in particular, might be. Bearing that in mind, our results contrast research that examined general LoC (e.g. [34]) but not necessarily that which examined specific LoC (e.g. [12]).

Another unexpected pattern is related to vaccine hesitancy which was almost unrelated to psychological control. Paradoxically, the single most effective measure that one can take personally in the face of global and overwhelming threat is not related to the preference for personal control or to the belief in possessing control. While we write this discussion, papers appear on a daily basis providing novel evidence about the psychological underpinnings related to vaccine intention. So, to the best of our current knowledge, mixed results are available, with some reporting that vaccine acceptance is positively [42] and some negatively related [37] to the external locus of control. There are also studies reporting a negative link with internal locus of control (e.g. [43]) and others indicating no link at all or an extremely weak link [44,45]. This makes control a variable that needs further investigation, possibly identifying key moderators, but ultimately not the best candidate to explain differences between realist and biased respondents.

Interestingly, the qualitative analyses revealed that risk perception related to vaccination side effects is a relevant topic associated with a reduced intention to get the COVID-19 vaccine. This suggests that in order to understand the differences between CRs and COs in terms of vaccine intention, it is fundamental to analyse how these two groups perceive the threat related to vaccination side effects. Indeed, it is plausible that CO participants may be optimistic not only about the risk of COVID-19 contraction but also about the risk of vaccination-related side effects. Finally, the open question analysis suggests that realists are more critical about the time needed to develop an effective and safe vaccine against COVID-19.

## 4. Study 3: relationship between comparative bias and vaccine intention—the role of perceived threat of COVID-19 illness and COVID-19 vaccine

Upon concluding that variables related to psychological control are not suitable explanations for the relationship between realism and vaccine intention, we searched for another possible mechanism.

To date, in most of the studies regarding comparative optimism in the context of the COVID-19 pandemic, researchers have concentrated on comparative optimism as an independent variable—they were looking for outcomes of it and not for its roots. But to understand the surprising finding that those who do not display comparative optimism for COVID-19 infection are less willing to get vaccinated, we decided to test the possible mechanisms of why comparative optimism emerges in the first place.

If CO is a reaction to a stressful situation (and possibly an adaptive one, or at least not inherently maladaptive; see [46]), then its strength should depend on the seriousness of the perceived threat.

Analogically, the intention to get vaccinated should also depend on the perceived level of threat from COVID-19, but with one important addition: the decision to vaccinate and to engage in other COVID-19 preventive measures also comes with possible negative consequences. We hypothesized that the final decision to get vaccinated must derive not only from the perceived threat from COVID-19 but also from the perceived threat from negative side effects of vaccination. Such a notion is supported by Study 2's qualitative analysis, in which those more opposed to vaccination were likely to mention fears and doubts regarding a vaccine's safety, a concern mirrored by realists.

Summing up, both realists and those less willing to vaccinate might share similar views on the severity of threats from COVID-19 illness and the COVID-19 vaccine: they might perceive illness as less dangerous and vaccines as more dangerous than optimists and vaccine-enthusiasts.

### 4.1. Method

Before the data collection, we pre-registered three hypotheses (see full pre-registration form: https://osf.io/387pt):

H1: CRs will hold a stronger belief that the development of COVID-19 vaccines was rushed too much (when compared with comparative optimists).
H2: CRs will have a lower COVID-19/vaccination fear ratio.
H3: Vaccine intention will correlate positively with COVID-19/vaccination fear ratio.

#### 4.1.1. Deviations from the pre-registered protocol

Post-data collection, we decided to change one feature of our pre-registered protocol in response to feedback from reviewers and readers. In our initial protocol we planned to compute the COVID-19/vaccination fear ratio, but for the sake of simplicity and consistency with the epidemiological literature, we decided to compute this variable as a difference instead of a ratio. Therefore, in the final form, the H3 reads ‘Vaccine intention will correlate positively with the difference between the fear of COVID-19 and the fear of COVID-19 vaccine (Threat_Difference_)’.

The analyses for the pre-registered variable can be found in the OSF folder and they lead to the same conclusions as analyses presented in the paper.

#### 4.1.2. Sample size justification, participants, procedure

Sample size justification was almost identical to that in Study 2. We strived to obtain the same power and the same alpha level to detect the same effect size. The only difference was the expected share of ‘realists’. Judging by the results from Study 2, we lowered the expected percentage of ‘realists’ to 20%, and to ensure the desired power we decided to recruit 1500 participants.

The final sample consisted of 1508 participants across 74 nationalities (563 males, 937 females, 3 non-disclosed, 5 missing data, *M*_Age_ = 25.69, min._Age_ = 18, max._Age_ = 65). For detailed information on sample demographics see the electronic supplementary material (https://osf.io/dp3n4).

As in Study 2, we excluded participants who were either vaccinated against COVID-19 or had been officially diagnosed with COVID-19. Moreover, we screened-out participants who took part in Study 2. Analogically to Study 2, *Prolific* sourced participants from all available countries, without quotas on demographics.

Data were collected online from 13 to 20 August 2021 from the *Prolific* panel. All questions used a ‘forced response’ option, which made proceeding to the next question impossible unless the participant provided a response for the current one. This study was approved by the local ethics committee. The questionnaire in the .qsf and .pdf file is publicly available in the electronic supplementary material (https://osf.io/4pd7v).

#### 4.1.3. Variables

##### 4.1.3.1. Comparative bias and vaccine intention

Comparative bias was assessed by the same three questions as in Study 1 and 2, inquiring about the perceived chance of COVID-19 infections for ‘me’, ‘peer’ and ‘countrymen’. The questions were answered on a 1 (absolutely impossible)–11 (quite certain) Likert-like scale. The answers were provided on a slider scale, with the default position as ‘1’.

The comparative index was also calculated as previously: *C*_Index_ *=* *(Q2* − *Q1)* *+* *(Q3* – *Q1)* and as previously, *C*_index_ was recoded into three categories: *C*_index_ = ‘0’ (CRs, comparative realists), *C*_index_ > 0 (COs, comparative optimists) and *C*_index_ < 0 (CPs, comparative pessimists).

The intention to get vaccinated was measured with the item: ‘I will take the vaccine for the coronavirus/SARS-CoV-2’.

Participants provided their answers on an 11-point scale (1 = *absolutely impossible*, 11 = *quite certain*). The answers were provided on a slider scale, with the default position as ‘1’.

##### 4.1.3.2. Belief in rushed vaccine development

This variable was measured by a single item: *How much do you agree with the statement: ‘The development of COVID-19 vaccines was rushed too much’?*

Participants were asked to provide answers on an 11-point scale (1 = *totally disagree*, 11 = *totally agree*).

##### 4.1.3.3. COVID-19 Disease and vaccine threat difference

In respect to COVID-19 disease threat estimates, the participants were first asked an open-ended question: *Please note down the first negative outcome of the COVID-19 infection that comes to your mind*.

In the next step, they were asked about the severity of this negative outcome:

Q1: How serious is this effect of the COVID-19 infection?

The answers were provided on an 11-point scale (1 = *not serious at all*, 11 = *most serious possible*).

Afterwards, they were asked about the perceived probability of this negative outcome:

Q2: What are the chances of suffering from the listed effects of the COVID-19 infection?

The answers were provided on an 11-point scale (1 = *almost impossible*, 11 = *almost certain*).

By multiplying the severity by probability, we computed a ‘negative expected value’ of COVID-19 disease: Threat_Disease_ = Q1 × Q2.

Regarding COVID-19 vaccination, we asked an analogical sequence of questions:

Vaccine open-ended threat: *Please note down the first negative outcome of the COVID-19 vaccination that comes to your mind*.

Q3: How serious is this side-effect of the COVID-19 vaccination?

Q4: What are the chances of suffering from this side-effect of the COVID-19 vaccination?

By multiplying the severity by probability, we computed a ‘negative expected value’ of COVID-19 vaccination: Threat_Vaccine_ = Q3 × Q4.

We computed a difference between threat from the disease and threat from vaccination:

$${\mathrm{Threat}}_{\mathrm{Difference}}={\mathrm{Threat}}_{\mathrm{Disease}}-{\mathrm{Threat}}_{\mathrm{Vaccine}.}.$$

### 4.2. Results

R programming language [38] was used to transform the data and JASP v. 0.14.1 [26] was used for the statistical analysis.

The distribution of categories of the *C*_Index_ was: ‘CRs’, 20.09% of the sample; ‘COs’, 70.16%; and ‘CPs’, 9.75%.

Again, the main effect was confirmed: comparative realists had significantly lower vaccine intention than comparative optimists: *U* = 198551.00, *r*_rb_ (1361) = 0.24, *p* < 0.001.

Mean vaccine intention for CRs was *M* *=* 5.26, s.d. = 3.61. For COs it was *M* *=* 6.78, s.d. = 3.59. The visualization of distributions are available at the OSF (https://osf.io/dp3n4).

#### 4.2.1. Confirmatory analyses

Since *C*_index_ and vaccine intention proved to have distributions significantly different from normal, we decided to test non-parametric statistics. Parametric analyses can be found in the OSF repository and they yield the same conclusions. To test H1 (realists will hold a stronger belief that the development of COVID-19 vaccines was rushed too much), we conducted an independent samples comparison with 'belief in rushed vaccine development' as a dependent variable and categorical C_Index_ (CRs/COs) as a grouping variable. Our hypothesis was confirmed—the Mann-Whitney test yielded significant results (*U* = 130034.00, *r*_rb_(1361) = −0.19, *p* < 0.001).

H2 (realists will have a lower Threat_Difference_) was also confirmed. An independent samples comparison with Threat_Difference_ as the dependent variable and categorical *C*_Index_ (CRs/COs) as a grouping variable indicated significant differences in the predicted direction (*U* = 190418.50, *r*_rb_ (1361) = 0.19, *p* < 0.001).

**Figure 5.**
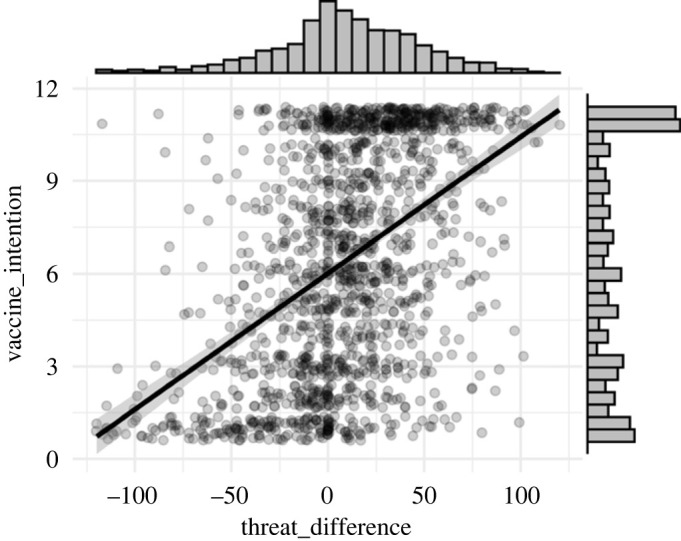
Correlation between Threat_Difference_ and vaccine intention along with the distribution plots of the two variables. Scatterplot points have been jittered, ribbons around regression line represents 96% CI.

To test H3 (vaccine intention will correlate positively with Threat_Difference_) we used Spearman's rank correlation, because the vaccine intention variable deviates from the assumption of normal distribution (figure 5).

The hypothesis was confirmed—Threat_Difference_ proved to be moderately correlated with vaccine intention (*rs*_1508_ = 0.49, *p* < 0.001) (figure 5).

Analogous to Study 2, we tested the hypotheses using three alternative operationalizations of CRs and COs: (i) CRs defined as *C*_index_ between ‘−1’ and ‘1’ and COs as *C*_index_ > 1; (ii) *C*_index_ computed as Risk_Peer_ – Risk_Me_, and (iii) *C*_index_ computed as Risk_Countrymen_ – Risk_Me_.

All three alternative analyses yielded the same conclusions—H1 and H2 were confirmed. The analyses can be found in the OSF folder (https://osf.io/skc5d/).

#### 4.2.2. Exploratory analyses

Our predictions were all confirmed. Lower COVID-19 disease–vaccination Threat_Difference_ is associated with both ‘realism’ and vaccine intention. Additionally, we identified one concrete and common concern that is more prevalent among comparative realists than comparative optimists, namely the concern about vaccine development being rushed too much.

**Figure 6.**
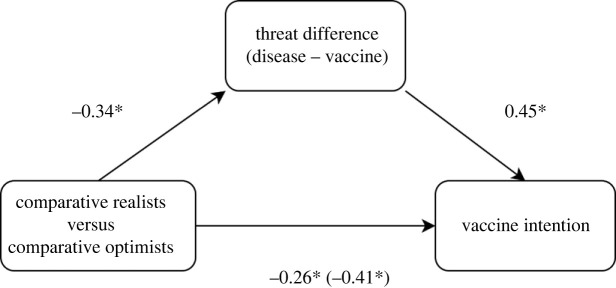
Path plot for mediation model with ‘vaccine intention’ as the dependent variable, categorical C_Index_ (CRs versus COs) as a predictor and Threat_Difference_ as a mediator.

In the next step, we decided to explore mediation models. The first model tested categorical *C*_Index_ (‘CRs’ coded as 0 versus ‘COs’ coded as 1) as a predictor, vaccine intention as a dependent variable and Threat_Difference_ as a mediator (figure 6). The mediation analysis (Delta method standard errors, ML estimator, standardized coefficients) indicated that there was a significant total effect (*Β* = −0.41, s.e. = 0.06, *p* < 0.001) and direct effect (*Β* = −0.26, s.e. = 0.06, *p* < 0.001) of categorical *C*_Index_ on vaccine intention. We detected a significant indirect effect of Threat_Difference_: *Β* = −0.15, s.e. = 0.03, *p* < 0.001. Total effect of *C*_Index_ on vaccine intention was positive, which means that comparative optimism (as opposed to realism) predicts higher vaccine intention. The model accounted for 23% of variance in vaccine intention, and the mediator Threat_Difference_ accounted for 37% of the total effect.

The second mediation model assumed that the categorical *C*_Index_ is an outcome variable. It was meant to represent the theoretical model in which engagement in various COVID-19 preventive strategies may lead an individual to be comparatively optimistic and engagement in preventive strategies is rooted in threat estimations.

Specifically, we tested and confirmed that the model that assumes Threat_Difference_ influences vaccine intention, which then influences the *C*_Index_ (CRs versus COs), is also empirically supported: the indirect effect of Threat_Difference_ on categorical *C*_Index_, mediated by the vaccine intention (*Β* = 0.09, s.e. = 0.02, *p* < 0.001) was significant. The direct effect was also significant: *Β* = 0.11, s.e. = 0.04, *p* = 0.01 and the total effect of Threat_Difference_ on *C*_Index_ was *Β* = 0.19, s.e. = 0.04, *p* < 0.001.

The model accounted for 6% of the variance of categorical *C*_Index_ and vaccine intention accounted for 45% of the total effect.

As the last exploratory analyses, we wanted to test whether Threat_Difference_ explains the variance of vaccine intention beyond the fear of the vaccines (Threat_Vaccine_).

To test this, we conducted a linear regression analysis, which included Threat_Vaccine_ as a part of the ‘null model’ and then inspected the significance of *R*^2^ change with the model including additional Threat_Difference_. We ran separate analyses for comparative optimists and comparative realists.

In the case of COs, the model consisting of just Threat_Vaccine_ accounted for 19% of the variance of vaccine intention. The model with additional Threat_Difference_ accounted for 23% of the variance and the *R*^2^ change was statistically significant: $R_{\mathrm{Change}}^{2}=0.04$, *F*_Change_ (1, 1055) = 60.87, *p* < 0.001.

In the case of CRs, Threat_Vaccine_ accounted for 16% of the variance, while adding Threat_Difference_ yielded 26% of explained variance. *R*^2^ change was significant, $R_{\mathrm{Change}}^{2}=0.10$, *F*_Change_ (1, 300) = 40.76, *p* < 0.001. An analogical analysis comparing models with Threat_Disease_ instead of Threat_Vaccine_ can be found in the OSF folder.

### 4.3. Discussion

The study provided evidence that realists and vaccine-hesitant people had at least two shared traits: they hold stronger beliefs about vaccines being developed too quickly and they assign different weights to threats from the COVID-19 disease and vaccine: vaccine-hesitant and comparative realists are less afraid of the disease and more afraid of the vaccine.

One plausible theoretical explanation for these commonalities comes from protection motivation theory (PMT [47]). In the PMT model, changes in attitudes and behaviours are driven by the fear of negative consequences of current behaviour. In this model, attitude or behaviour change is caused by individuals' perception of three domains:

(1) severity of negative consequences of maintaining the current state,
(2) probability of negative consequences of maintaining the current state, and
(3) efficacy of the considered alternative.

When it comes to vaccination, one additional factor seems to be at play—fear of the negative outcomes of the vaccine itself, and this is where the recent expansion of the PMT is needed [48]. In the PMT expansion, a fourth and fifth dimension are considered:

(4) severity of negative consequences of the alternative behaviour, and
(5) probability of negative consequences of the alternative behaviour.

In this framework, when an individual considers any preventive, anti-COVID-19 measure (be it vaccination or mask wearing), their final decision would be positive if: (i) they are convinced that the negative outcomes of changing nothing and living as ‘usual’ will be dreadful, (ii) they are quite sure that they will face these consequences, (iii) they believe preventive measures can actually work, and (iv) they believe that the preventive measures bear no significant risk to themselves.

Extended PMT theory can also explain why vaccine intention mediates the relationship between Threat_Difference_ and comparative bias. In that framework, people become comparative optimists due to the measures they take, and they take these measures because they believe that they can outweigh the potential harm from COVID-19. Comparative realists, on the other hand, are aware of their disengagement, and this disengagement might be born out of the equilibrium of threats they perceive from the disease and the cure.

## 5. General discussion

Comparative optimism is a robust phenomenon. The bias proved to be present inter-contextually [46], and since the first theoretical works in the 1980s, it is still considered a replicable and practically significant effect. Furthermore, the bias has been successfully discovered by multiple research teams in many settings during the COVID-19 pandemic [49–51]. But do social psychologists have a firm understanding of why this bias occurs and its consequences?

As with many other collective irrationalities, we can too often be taken in by the ‘rational = desirable’ narrative. In such a narrative we implicitly or explicitly assume that the most desirable state would be ‘unbiased’, and, if the examined population fails to adhere to this pattern, we conclude that the cognitive processes we examine are somewhat ‘flawed’. In the presented studies, we concluded that those who are ‘unbiased’ more often abstain from taking one of the most (if not *the* most) effective, evidence based and affordable actions that could protect them from deadly threat. A seemingly ‘rational’ mental approach to the issue of COVID-19 contraction is related to a more irrational response to that threat—namely not getting vaccinated.

In the mini meta-analysis and two pre-registered studies, we discovered that those who express either comparative pessimism or optimism have a higher intention to get vaccinated for COVID-19 than those who are unbiased. The relationship of comparative pessimism to pro-health behaviour seems more intuitive, and the positive relationship of comparative optimism comes as a surprise, but our discovery is not isolated in that regard [52].

In Study 2, we found no evidence of a relationship between psychological control and comparative optimism with vaccine intention.

In Study 3 we found a common denominator of people who are realists and who have a lower vaccine intention. It turned out that both phenomena are related to lower COVID-19 Threat_Difference_ (Threat_Disease_ − Threat_Vaccine_). Furthermore, in line with the extended protection motivation theory (PMT [47,48]), the trade-off between risks of the disease and risks of the vaccine proved to predict being unbiased, and this relationship is partly mediated by vaccine intention.

Our studies present evidence that counters the ‘rational = desirable’ narrative, but that could lead into another trap: assuming that it is irrationalities and biases that help us cope more effectively. We think that such a narrative can be an equally false over-simplification and our studies offer more compelling explanations.

Collective irrationalities, such as comparative optimism may neither enhance nor hamper our coping abilities. They may, in turn, be a by-product of ongoing coping processes, possibly leading to greater protection (in the case of our studies, vaccination against COVID-19). From the perspective of our studies, it is clear that we might wrongfully ascribe a causal role to these biases.

While one might think that comparative optimism may cause reckless behaviour, such as refusal to vaccinate, Study 3 suggests another plausible alternative mechanism: Threat_Difference_ might be the reason for stronger or weaker vaccine intention (along with many other factors; see [43,53]) and comparative optimism might be a result of knowing one's own efforts, such as vaccination. In fact, a recent experimental study [52] provides evidence that being more aware of one's own self-protective effort enhances comparative optimism.

It is also noteworthy that comparative biases may arise in part from a lack of information about the comparative target, and that providing people with information about the comparative target diminishes the bias [54]. Accordingly, the comparative optimists in our study may have lacked information about the preventive behaviour of others.

The case of the relationship between comparative optimism and constructive pro-health behaviour is complex. On the one hand, we have evidence for both the benefits and drawbacks of CO [55]. On the other hand, CO may be the result rather than the cause of pro-health behaviour. Clearly there are many contextual factors involved and we should discard the overly simplistic view of an inherently beneficial or inherently harmful nature of comparative optimism (which also might be the case for many other collective irrationalities).

Our paper presents a pre-registered and high-powered line of research, which addresses differences between comparative optimists and the ‘unbiased’—a category of individuals that has most often been either left undiscussed or barely mentioned in previous studies regarding CO. Examining the bias from the perspective of the unbiased and using a mixed method approach that combined theory-driven hypotheses with a bottom-up strategy, thus giving a voice to participants, offered the opportunity to enrich theoretical knowledge on comparative bias and led to the surprising discovery that being unbiased can be related to a less pro-health attitude.

### 5.1. Limitations and future directions

The main limitation of our study is the lack of behavioural measures. This was a result of an early stage of our research project, which took place before COVID-19 vaccines were available. For that reason, we gathered data only about vaccine intention. In follow-up studies the vaccines were available but we decided to examine the intention of the yet unvaccinated to ensure the direct comparability of follow-up studies with the studies from a mini meta-analysis. This limitation leads to another one—at the time of Study 2 and especially Study 3, the number of unvaccinated was shrinking and we can expect that they might differ from the general population in many ways (for example, from study to study, we observed the diminishing share of ‘realists’). This constitutes a limit for the generalization of our conclusions.

The future direction of research regarding the differences between unbiased and comparative optimists should concentrate on actual behaviours rather than intentions or declarations. Moreover, future studies should enhance the scope of generalization by investigating more representative samples.

Another limitation is the possibility of an alternative explanation of our results. We interpret the results of Study 3 in the light of the extended PMT theory, assuming that the relationship between predicted outcomes of falling ill and getting vaccinated leads to engagement or disengagement with vaccination, which it turn results in them feeling superior (comparatively optimistic) or similar (comparatively realistic) to others.

But an alternative is probable. Following Gigerenzer's theory of ‘fast and frugal heuristics' [56], people can often make more ecologically valid decisions when they follow heuristics, without engaging in deep, analytical processes.

Perhaps people who chose the ecologically rational option to take the vaccine did so because they followed their intuition/shortcuts when making the decision. By doing so, they estimated the trade-offs between the disease and vaccine in line with the mainstream message (media, experts and authorities). If these individuals followed intuition in this respect, they may also be more prone to the default bias, namely optimistic bias. On the other hand, people who engage in processing the information more reflectively might end up being more sceptical towards vaccination and also less prone to the optimistic bias.

These alternative explanations could be empirically tested—if pro-vaccine attitudes could be ascribed to using more ‘fast and frugal heuristics’, people more sceptical of the vaccines should be able to recall more information about vaccines (regardless of their epistemic status) and provide more elaborate explanations for their stance.

As a general direction for future research on comparative biases, we advocate for considering a categorical approach to measuring biases—individuals who do not exhibit a bias should be treated as a separate category, especially when empirical results would indicate a substantial inflation of scores signalling a lack of bias (a similar inflation has been identified in the case of dehumanization—see [57], p. 12). Alternatively, if one decides to treat comparative bias as a continuous scale, a nonlinear relationship should be investigated. If comparative biases can have two directions, it is reasonable to expect that different directions might have different correlations.

## Data Availability

All data, reproducible analyses files and study materials can be accessed in Open Science Framework public repository: https://osf.io/skc5d/.

## Footnotes

1 Please note that comparative optimism, pessimism or realism should not be conflated with the accuracy of judgements. On an individual level, it is almost impossible to determine whether one is right or wrong in their comparative judgements of risk. What we can detect and what serves as the focus of this paper is the presence or absence of the pessimistic/optimistic tendencies in self–others comparisons.
